# Advanced lung cancer inflammation index is associated with mortality in critically ill patients with acute kidney injury

**DOI:** 10.1016/j.clinsp.2026.101156

**Published:** 2026-09-19

**Authors:** Qingquan Liu, Yuncheng Zhang, Yuan Xia, Binfeng Li

**Affiliations:** a Department of Nephrology, Tongji Hospital, Tongji Medical College, Huazhong University of Science and Technology, Wuhan, China; b Department of the Second Clinical College, Tongji Hospital, Tongji Medical College, Huazhong University of Science and Technology, Wuhan, China; c Department of Centralized Intravenous Drug Dispensing Center, Hubei Cancer Hospital, Tongji Medical College, Huazhong University of Science and Technology, Wuhan, China; d Department of Thoracic Surgery, Hubei Cancer Hospital, Tongji Medical College, Huazhong University of Science and Technology, Wuhan, China

**Keywords:** Advanced lung cancer inflammation index, Acute kidney injury, Nutrition, Inflammation, Intensive care unit, Mortality, Biomarker

## Abstract

**Background:**

Nutritional and inflammatory status are closely associated with outcomes in Acute Kidney Injury (AKI). This study investigated the association between the Advanced Lung cancer Inflammation index (ALI), a composite nutrition-inflammation marker, and short-term mortality in critically ill patients with AKI.

**Methods:**

We conducted a retrospective cohort study using the MIMIC-IV database. A total of 1887 adult ICU patients with AKI were included. ALI was calculated as body mass index × serum albumin / neutrophil-to-lymphocyte ratio. The primary outcome was all-cause in-hospital mortality, and the secondary outcome was 90-day mortality. Kaplan-Meier curves, Cox proportional hazards models, restricted cubic spline analysis, and subgroup analyses were performed.

**Results:**

The median age was 68.0-years, and 62.4% of patients were male. In-hospital and 90-day mortality rates were 25.2% and 33.9%, respectively. Patients in the lowest ALI tertile had significantly higher cumulative mortality than those with higher ALI. After full adjustment, higher ln(ALI) was independently associated with lower in-hospital mortality (HR = 0.86; 95% CI 0.79–0.93; p < 0.001) and lower 90-day mortality (HR = 0.81; 95% CI 0.75–0.87; p < 0.001). Restricted cubic spline analysis showed an approximately linear inverse association between ALI and in-hospital mortality.

**Conclusions:**

Lower ALI was associated with higher short-term mortality in critically ill patients with AKI, but this association was substantially attenuated after full adjustment and was largely confined to non-septic AKI. This retrospective single-center analysis supports an association but does not establish clinical utility; prospective multicenter external validation is needed before ALI can be recommended for risk stratification.

## Highlights

• Lower ALI was associated with higher short-term mortality, but this association was attenuated after full adjustment.
• ALI showed a linear inverse association with in-hospital mortality.
• Lower ALI predicted higher 90-day mortality in critically ill AKI.

## Introduction

Acute Kidney Injury (AKI) is a common and serious complication in critically ill patients, associated with prolonged hospitalization, high healthcare costs, and substantial short-term mortality.^1^^,^^2^ Despite advances in renal replacement therapy and supportive care, the mortality rate of severe AKI remains high, and early identification of high-risk patients is crucial for risk stratification and clinical decision-making.^3^

Inflammation and malnutrition are two interconnected pathophysiological processes that play key roles in the development and progression of AKI.^4^^,^^5^ In critically ill patients, systemic inflammation can exacerbate kidney injury, while pre-existing or acquired malnutrition impairs immune function and tissue repair.^6^^,^^7^

Several inflammatory biomarkers, such as Neutrophil-to-Lymphocyte Ratio (NLR), C-reactive protein and systemic inflammation response index, have been shown to predict adverse outcomes in AKI.^8^ Similarly, malnutrition is closely related to an increased in-hospital mortality rate in ICU patients with AKI.^9^^,^^10^ Low Body Mass Index (BMI) and hypoalbuminemia are associated with increased mortality in AKI populations.^11^^,^^12^ However, most existing studies have evaluated these factors in isolation, and a simple integrated score reflecting both inflammation and nutrition is still lacking for AKI patients.

The Advanced Lung cancer Inflammation Index (ALI) was originally developed to predict prognosis in patients with non-small cell lung cancer.^13^ It combines BMI, serum albumin, and NLR into a single formula: BMI×Alb/NLR. Recent studies have extended its application to other critical illnesses and chronic disease populations, including Crohn's disease, coronary artery disease, heart failure, and type 2 diabetes.^14^^,^^15^ For example, among critically ill patients with heart failure, lower ALI was independently associated with higher long-term mortality.^14^ Previous studies have examined the prognostic value of ALI in sepsis-associated AKI,^16^ but evidence in broader critically ill AKI populations remains limited. Furthermore, ALI has not been validated in non-cancer critical illness populations beyond the MIMIC-IV database, and the optimal cutoff values may differ across clinical contexts. Given that AKI shares inflammatory and nutritional derangements with other critical illnesses, ALI may also be associated with mortality in this population.

Therefore, the aim of this study was to investigate whether baseline ALI is independently associated with in-hospital and 90-day mortality in critically ill patients with AKI, using a large, publicly available intensive care database. We also sought to evaluate whether the association is linear, to assess its consistency across major clinical subgroups, and to discuss the incremental prognostic value of ALI beyond established risk scores.

## Participants and methods

### Data source and study population

This retrospective cohort study used data from the Medical Information Mart for Intensive Care IV (MIMIC-IV) database, which contains de-identified clinical data of patients admitted to the Intensive Care Units (ICUs) at Beth Israel Deaconess Medical Center from 2008 to 2019.^17^ The first author, Qingquan Liu, obtained formal authorization to extract data after completion of the required training course (certification number: ID: 51,474,658). The study followed the STROBE reporting guideline for observational studies. PostgreSQL software was used to access the MIMIC-IV database, and all research data were retrieved using Structured Query Language (SQL) code. The project was approved was approved by the Institutional Review Board of the Massachusetts Institute of Technology. Because the database contains de-identified publicly available data, the requirement for individual informed consent was waived.

We included adult patients (age ≥ 18-years) with a diagnosis of AKI at ICU admission, identified based on the Kidney Disease Improving Global Outcome (KDIGO) criteria (Khwaja, 2012) were selected as research subjects. Both SCr and urine volume were used to categorize the stages of AKI. For patients with multiple ICU admissions, only the first admission was considered. Exclusion criteria were: 1) Missing data on BMI, serum albumin, neutrophil count, or lymphocyte count; 2) ICU stay less than 48-hours; and 3) Pre-existing end-stage kidney disease on chronic dialysis. Figure S1 depicts the patient selection process, with 1887 adult patients meeting the criteria. Because the MIMIC-IV database contains de-identified public data and access requires completion of data-use training, informed consent was waived by the database oversight bodies.

### Outcome measures

The primary outcome was all-cause in-hospital mortality. The secondary outcome was all-cause mortality within 90-days of ICU admission. Survival status and follow-up time were obtained from hospital records and linked state death records in MIMIC-IV. Death dates more than one year after hospital discharge are censored as part of the MIMIC-IV de-identification process.

### Covariates

Demographic variables included age and sex, and Body Mass Index (BMI) was recorded. Vital signs and laboratory variables were obtained from the first measurement after ICU admission. Comorbidities included hypertension, diabetes mellitus, myocardial infarction, cancer, hyperlipidaemia, depression, chronic liver disease, chronic pulmonary disease, rheumatic disease, peripheral vascular disease, and iron-deficiency anaemia. Disease severity was assessed using the SOFA score and the Charlson Comorbidity Index (CCI). In addition, we extracted several clinically important variables from MIMIC-IV: KDIGO AKI stage, 24-hour urine output and oliguria, Renal Replacement Therapy (RRT), mechanical ventilation, vasopressor exposure, peak lactate within 24-hours, 24-hour fluid balance, and nutritional support. ALI was calculated as ALI=BMI(kg/m²)×serumalbumin(g/dL)/NLR, where NLR is the ratio of absolute neutrophil count to absolute lymphocyte count,^18^ measured from the first blood sample collected within 24-hours of ICU admission.

### Risk of bias assessment

The risk of bias was assessed using the Risk of Bias In Non-randomized Studies of Interventions (ROBINS-I) tool, which evaluates seven domains. The overall risk of bias was judged as moderate, primarily due to the retrospective observational design, the use of ICD codes for AKI identification rather than KDIGO criteria, and the absence of several important clinical confounders. The domain-specific judgements are summarized in Supplementary Table 1.

### Statistical analysis

Continuous variables with normal distributions were presented as mean ± Standard Deviation (SD), while non-normally distributed variables were expressed as median (interquartile range). Categorical variables were reported as numbers and percentages. Baseline characteristics were compared across ALI tertiles using analysis of variance or Kruskal-Wallis tests for continuous variables and chi-square tests for categorical variables, as appropriate. Kaplan-Meier curves with log-rank tests were used to compare cumulative mortality between ALI tertiles. Cox proportional hazards regression models were used to estimate the association between ALI and mortality.

ALI was natural-log transformed [ln(ALI)] and modeled as a continuous variable and as tertiles for both outcomes; the log transformation provided a better model fit than untransformed ALI (lower Akaike information criterion; ΔAIC = 28.78 for in-hospital and 65.79 for 90-day mortality). Covariate selection was guided by standard epidemiological criteria for confounding (associated with both ALI and mortality and not on the causal pathway); accordingly, the constituent components of ALI (BMI, serum albumin, and the neutrophil-to-lymphocyte ratio) were not included as covariates, and lactate, KDIGO stage, and oliguria, which may represent confounding or mediation, were conservatively treated as potential confounders. Four models with progressive adjustment were constructed: Model 1, unadjusted; Model 2, adjusted for age and sex; Model 3, additionally adjusted for disease-severity and baseline physiological markers (SOFA score, Charlson Comorbidity Index, lactate, KDIGO stage, oliguria, heart rate, glucose, blood urea nitrogen, and white blood cell count); and Model 4, further adjusted for organ-support interventions (RRT, mechanical ventilation, vasopressor use, fluid balance, and nutritional support).

Restricted cubic splines with three knots were fitted to examine potential nonlinearity. Subgroup analyses were performed by age (18–65 vs. > 65-years), sex, BMI (< 25, 25–30, and ≥ 30 kg/m^2^), hypertension, diabetes, and hyperlipidemia, and interaction terms were tested in the Cox models. All analyses were performed using R version 4.2.1; a two-tailed p < 0.05 was considered statistically significant.

The proportional hazards assumption was formally tested using Schoenfeld residuals. Global and covariate-specific tests revealed that the proportional hazards assumption was violated for the SOFA score (p < 0.001) and, to a lesser degree, for the Charlson Comorbidity Index and ln(ALI) (p < 0.05). Because the SOFA score is inherently a time-varying severity measure, we retained it in the models but additionally performed stratified, competing-risk, and multiple-imputation sensitivity analyses to assess the robustness of the findings (see below).

In addition, standardized differences were calculated for all baseline characteristics across ALI tertiles to complement p-values, as large sample sizes may yield statistically significant differences that are not clinically meaningful. An absolute standardized difference > 0.10 was considered indicative of a meaningful imbalance.

## Results

### Baseline characteristics

A total of 1,887 critically ill patients with AKI were included in the final analysis. The median age of the overall cohort was 68.0-years (IQR 58.0–77.0), and 62.4% were male. In-hospital and 90-day mortality rates were 25.2% and 33.9%, respectively. Based on tertiles of ALI, patients were categorized into three groups: T1 (ALI < 6.05, n = 629), T2 (ALI 6.05–14.92, n = 629), and T3 (ALI ≥ 14.92, n = 629). Significant differences were observed across the three groups in multiple demographic, laboratory, and clinical parameters. Compared with patients in the highest ALI Tertile (T3), those in the lowest Tertile (T1) were older (median age, 70.0 vs. 67.0 years; p = 0.01) and had lower BMI (median, 26.5 vs. 31.1 kg/m^2^; p < 0.001). The T1 group also had higher leukocyte count (median, 15.5 vs. 10.7 × 10^9/L; p < 0.001), NLR (median, 22.9 vs. 4.0; p < 0.001), creatinine (median, 1.5 vs. 1.3 mg/dL; p < 0.001), and BUN (median, 32.0 vs. 22.0; p < 0.001). In addition, patients in T1 had lower prevalence of hyperlipidemia and lower albumin, sodium, and calcium levels. With respect to disease severity and outcomes, patients in T1 had higher SOFA scores (median, 7.0 vs. 5.0; p < 0.001), higher CCI (median, 6.0 vs. 5.0; p = 0.011), higher in-hospital mortality (35.0% vs. 19.1%; p < 0.001), and higher 90-day mortality (46.6% vs. 24.8%; p < 0.001) than patients in T3. No significant differences were observed in hospital length of stay (median, 16.5 vs. 15.6 days; p = 0.872) or ICU length of stay (median, 5.7 vs. 5.3 days; p = 0.861). Standardized Mean Differences (SMD) for all baseline variables are reported in Table 1; variables with an absolute SMD > 0.10 (including NLR, BMI, leukocyte count, albumin, and SOFA score) confirmed clinically meaningful imbalances across ALI tertiles.

**Table 1:** Baseline characteristics of patients, divided according to the advanced lung cancer inflammation index.

Baseline characteristics	Total (n = 1887)	T1 (n = 629)	T2 (n = 629)	T3 (n = 629)	p-value	SMD
Age, median (IQR), year	68.0 [58.00,77.0]	70.0 [60.0, 78.0]	68.0 [58.0, 78.0]	67.0 [56.0, 76.0]	0.01	0.155
Female (n, %)	709 (37.6)	230 (36.6)	242 (38.5)	237 (37.7)	0.782	0.019
BMI median, (IQR), kg/m²	28.9 [24.9,34.2]	26.5 [23.0, 31.8]	28.7 [25.1, 33.3]	31.1 [27.1, 36.7]	<0.001	0.620
Heart rate, median (IQR), bpm	85.8 [75.1, 99.5]	90.0 [77.9, 104.0]	83.7 [73.3, 96.5]	83.8 [75.0, 97.7]	<0.001	0.302
SpO_2_, median (IQR), %	96.9 [95.2, 98.3]	96.8[95.0, 98.3]	96.9 [95.3, 98.4]	96.9 [95.3, 98.3]	0.462	0.089
SBP, median (IQR), mmHg	111.2 [103.6120.3]	111.3 [101.3, 121.8]	111.7 [104.3, 120.7]	110.1 [104.4, 118.5]	0.489	0.079
DBP, median (IQR), mmHg	56.9 [51.1, 62.6]	57.1 [51.2, 62.7]	57.0 [52.0, 62.5]	56.6 [50.8, 62.5]	0.454	0.089
**Medical history**						
Hypertension (n, %)	603 (31.9)	198 (31.5)	193 (30.7)	209 (33.2)	0.612	0.025
Diabetes mellitus Type 2 (n, %)	655 (34.7)	192 (30.5)	216 (34.3)	244 (38.8)	0.009	0.083
Diabetes mellitus Type 1 (n, %)	35 (1.9)	10 (1.6)	12 (1.9)	10 (1.6)	0.881	0.003
Myocardial infarct (n, %)	389 (20.6)	122 (19.4)	141 (22.4)	123 (19.6)	0.327	0.030
Cancer (n, %)	263 (13.9)	107 (17.0)	88 (14.0)	65 (10.3)	0.003	0.067
Hyperlipidaemia (n, %)	790 (41.9)	222 (35.3)	275 (43.7)	290 (46.1)	<0.001	0.108
Depression (n, %)	251 (13.3)	70 (11.1)	85 (13.5)	93 (14.8)	0.15	0.037
Liver disease (n, %)	36 (1.9)	8 (1.3)	13 (2.1)	12 (1.9)	0.523	0.008
Chronic pulmonary disease (n, %)	95 (5.0)	33 (5.2)	33 (5.2)	26 (4.1)	0.571	0.011
Rheumatic disease (n, %)	171 (9.1)	46 (7.3)	59 (9.4)	63 (10.0)	0.213	0.027
Peripheral vascular disease (n, %)	71 (3.8)	29 (4.6)	21 (3.3)	18 (2.9)	0.228	0.017
Iron deficiency anaemia (n, %)	97 (5.1)	37 (5.9)	29 (4.6)	28 (4.5)	0.442	0.014
**Laboratory tests**						
Haemoglobin, median (IQR), g/dL	10.2 [8.4, 12,2]	10 [8.3, 12.0]	10.2 [8.4, 12.1]	10.3 [8.5, 12.5]	0.31	0.106
Erythrocyte, median (IQR), × 10^12^/L	3.5 [2.8, 4.2]	3.4 [2.8, 4.1]	3.4 [2.8, 4.1]	3.6 [2.9, 4.3]	0.582	0.111
MCV, median (IQR), fL	92.0 [88.0, 97.0]	92.0 [88.0, 97.0]	92.0 [88.0, 97.0]	92.0 [88.0, 97.0]	0.831	0.054
MCH, median (IQR), pg	29.8 [28.2, 31.4]	29.7 [28.0, 31.3]	30.1 [28.5, 31.6]	29.7 [28.2, 31.4]	0.419	0.116
MCHC, median (IQR), %	32.2 [31.2, 33.3]	32.2 [31.1, 33.2]	32.4 [31.3, 33.4]	32.2 [31.1, 33.2]	0.347	0.148
RDW, median (IQR), %	14.8 [13.7, 16.7]	15.2 [13.9, 17.2]	14.7 [13.5, 16.5]	14.6 [13.5, 16.3]	0.14	0.263
Leucocyte, median (IQR), × 10^9^/L	12.2 [8.5, 17.9]	15.5 [10.2, 21.8]	12.2 [8.5, 17.4]	10.7 [7.4, 14.5]	<0.001	0.412
Lymphocytes, median (IQR), × 10^9^/L	9.0 [5.0, 14.5]	4.0 [2.5, 5.1]	9.1 [7.4, 11.6]	17.6 [13.4, 23.3]	<0.001	2.072
Neutrophils, median (IQR), %	80.0 [73.0, 85.9]	87.8 [83.9, 91.0]	80.6 [77.0, 84.0]	70.8 [62.2, 76.8]	<0.001	1.643
NLR (median [IQR])	8.89 [5.05,17.24]	22.9 [16.5, 35.9]	8.8 [6.7, 10.9]	4.0 [2.6, 5.6]	<0.001	1.682
Monocytes, median (IQR), %	6.0 [3.6, 9.0]	4.4 [3.0, 6.9]	6.7 [4.2, 9.2]	7.6 [4.3, 10.5]	<0.001	0.641
PT, median (IQR), s	14.9 [12.9, 18.7]	15.4 [13.2, 19.6]	14.8 [12.9, 18.4]	14.6 [12.8, 17.9]	0.653	0.190
INR, median (IQR)	1.4 [1.2, 1.7]	1.4 [1.2, 1.8]	1.4 [1.2,1.7]	1.3 [1.2,1.6	0.634	0.211
Potassium, median (IQR), mEq/L	4.3 [3.9, 4.8]	4.3 [3.8, 4.9]	4.3 [3.9, 4.9]	4.3 [3.9, 4.8]	0.461	0.050
Glucose, median (IQR), mEq/L	136.0 [109.5, 184.0]	143.0 [111.0, 196.0]	138.0 [112.0, 190.0]	131.0 [106.0, 170.5]	<0.001	0.177
Creatinine, median (IQR), mg/dL	1.3 [1, 1.9]	1.5 [1.10, 2.10]	1.3 [1.0, 1.8]	1.3 [1.0, 1.7]	<0.001	0.216
BUN, median (IQR), mEq/L	27.0 [18.0, 43.0]	32.0 [21.0, 50.0]	27.0 [18.0, 42.0]	22.0 [16.0, 36.0]	<0.001	0.430
Albumin, median (IQR), g/dL	3.2 [2.6, 3.4]	2.7 [2.3, 3.1]	3.0 [2.6, 3.4]	3.2 [2.8, 3.6]	<0.001	0.868
Sodium, median (IQR), mEq/L	138.0 [135.0, 141.0]	137.0 [134.0, 141.0]	138.0 [135.0, 141.0]	138.0 [135.0, 141.0]	0.006	0.141
Calcium, total, median (IQR), mg/dL	8.3 [7.8, 8.8]	8.1 [7.6, 8.7]	8.3 [7.8, 8.8]	8.4 [7.9, 8.9]	0.003	0.280
Alanine aminotransferase, U/L	29.0 [16.0, 69.0]	32.0 [16.2, 81.3]	29.0 [16.0, 72.0]	27.0 [15.0, 56.0]	0.547	0.094
Aspartate aminotransferase, U/L	46.4 [26.4, 111.2]	49.0 [27.0, 124.0]	46.0 [25.0, 115.0]	45.5 [25.0, 91.0]	0.755	0.112
Alkaline phosphatase, U/L	83.5 [61.0, 120.0]	94.0 [66.0, 137.0]	80.0 [59.0, 117.3]	78.0 [59.0, 107.0]	0.234	0.290
Total bilirubin, mg/dL	0.7 [0.4, 1.5]	0.8 [0.4, 1.7]	0.7 [0.4, 1.4]	0.7 [0.4, 1.2]	0.773	0.183
Lactate, median (IQR), mmol/L	2.5 [1.6, 4.3]	2.8 [1.8, 4.9]	2.5 [1.6, 4.3]	2.2 [1.5, 3.7]	<0.001	0.225
**Disease severity**						0.086
KDIGO stage 3 (n, %)	462 (24.5)	182 (28.9)	152 (24.2)	128 (20.3)	0.020	
Urine output (24 h), median (IQR), mL	1350 [815, 2185]	1200 [610, 1995]	1345 [805, 2170]	1515 [1010, 2380]	<0.001	0.224
Oliguria (n, %)	667 (35.3)	257 (40.9)	212 (33.7)	198 (31.5)	0.001	0.094
SOFA score, median (IQR)	6.0 [4.0, 10.0]	7.0 [5.0, 11.0]	8.5 [4.0, 11.0]	7.0 [4.0, 9.0]	<0.001	0.301
Charlson comorbidity index, median (IQR)	6.0 [4.0, 8.0]	6.0 [4.0, 8.0]	6.0 [4.0, 8.0]	5.0 [3.0, 8.0]	0.011	0.172
**Organ support and interventions**						
Renal replacement therapy	148 (7.8)	57 (9.1)	52 (8.3)	39 (6.2)	0.150	0.029
Mechanical ventilation	1133 (60.0)	370 (58.8)	382 (60.7)	381 (60.6)	0.745	0.019
Vasopressor use	1076 (57.0)	381 (60.6)	348 (55.3)	347 (55.2)	0.088	0.054
Fluid balance (24 h)	1146 [−924, 4250]	1514 [−842, 4555]	1042 [−900, 4104]	981 [−1100, 3915]	0.050	0.110
Nutritional support	55 (2.9)	25 (4.0)	16 (2.5)	14 (2.2)	0.145	0.017
**Outcome**						
Length of hospital stay, median (IQR), days	16.2 [9.1, 26.8]	16.5 [9.1, 25.8]	16.0 [9.6, 27.0]	15.6 [8.9, 27.7]	0.872	0.087
Length of ICU stay, median (IQR), days	5.5 [2.9, 10.8]	5.7 [3.1, 10.2]	5.4 [2.8, 10.9]	5.3 [3.0, 11.1]	0.861	0.051
In-hospital mortality (n, %)	476 (25.2)	220 (35.0)	136 (21.6)	120 (19.1)	<0.001	0.159
90-day mortality (n, %)	639 (33.9%)	293 (46.7)	190 (30.2)	156 (24.8)	<0.001	0.218

BMI, Body mass index; SOFA; Sequential Organ Failure Assessment; ALI, Advanced Lung cancer Inflammation index; BUN, Blood Urea Nitrogen; SBP, Systolic Blood Pressure; DBP, Diastolic Blood Pressure; ICU, Intensive Care Unit; INR, International Normalized Ratio; MCH, Mean Corpuscular Haemoglobin; MCHC, Mean Corpuscular Haemoglobin Concentration; MCV, Mean Corpuscular Volume; NLR, Neutrophil-to-Lymphocyte Ratio; PT, Prothrombin Time; RDW, Red Blood cell Distribution Width; SOFA, Sequential Organ Failure Assessment; SpO_2_, Pulse Oxygen Saturation; IQR, Interquartile Range. SMD, Standardized Mean Difference; an absolute SMD > 0.10 indicates a meaningful imbalance between groups.

### Association between ALI and in-hospital mortality

Univariate Cox regression analysis was performed to identify factors associated with in-hospital mortality (Supplementary Table 2). In line with the primary study objective, we focused on the prognostic value of ALI and its individual components. Higher NLR (HR = 1.01, 95% CI 1.01–1.02; p < 0.001) was associated with increased mortality risk, whereas higher BMI was not statistically significant (HR = 0.99, 95% CI: 0.98–1.00; p = 0.127). Higher serum albumin was associated with lower mortality (HR = 0.79, 95% CI: 0.68–0.91; p = 0.001). ALI as a composite index was significantly associated with in-hospital mortality (Table 2).

**Table 2:** Association between ALI and in-hospital mortality.

Model		ln(ALI)	ALI tertiles			P for trend
			T1	T2	T3	
Model 1	HR (95% CI)	0.79 (0.73‒0.86)	Reference	0.61 (0.49, 0.75)	0.52 (0.42, 0.65)	<0.001
	p	<0.001		<0.001	<0.001	
Model 2	HR (95% CI)	0.80 (0.74‒0.87)	Reference	0.61 (0.49, 0.75)	0.53 (0.42, 0.66)	<0.001
	p	<0.001		<0.001	<0.001	
Model 3	HR (95% CI)	0.98 (0.89‒1.08)	Reference	0.83 (0.65‒1.07)	0.85 (0.65‒1.12)	0.187
	p	0.665		0.144	0.244	
Model 4	HR (95% CI)	0.97 (0.88, 1.07)	Reference	0.82 (0.64‒1.05)	0.86 (0.66‒1.12)	0.199
	p	0.541		0.112	0.268	

ALI, Advanced Lung cancer Inflammation Index; CI, Confidence Interval; HR, Hazard Ratio; ln, Natural logarithm.

Model 1: Unadjusted.

Model 2: Adjusted for age and sex.

Model 3: Model 2 + SOFA score, Charlson Comorbidity Index, lactate, KDIGO stage, oliguria, heart rate, glucose, blood urea nitrogen, and white blood cell count.

Model 4: Model 3 + RRT, mechanical ventilation, vasopressor use, fluid balance, and nutritional support.

Kaplan-Meier analysis indicated that the cumulative incidence of in-hospital mortality was significantly higher in critically ill AKI patients with lower ALI (log-rank test p < 0.001; Fig. 1). Patients who experienced in-hospital death or 90-day mortality exhibited a significantly lower ALI compared with survivors (both p < 0.001; Fig. S2); These differences remained statistically significant after Bonferroni correction for the two comparisons.

**Fig. 1:**
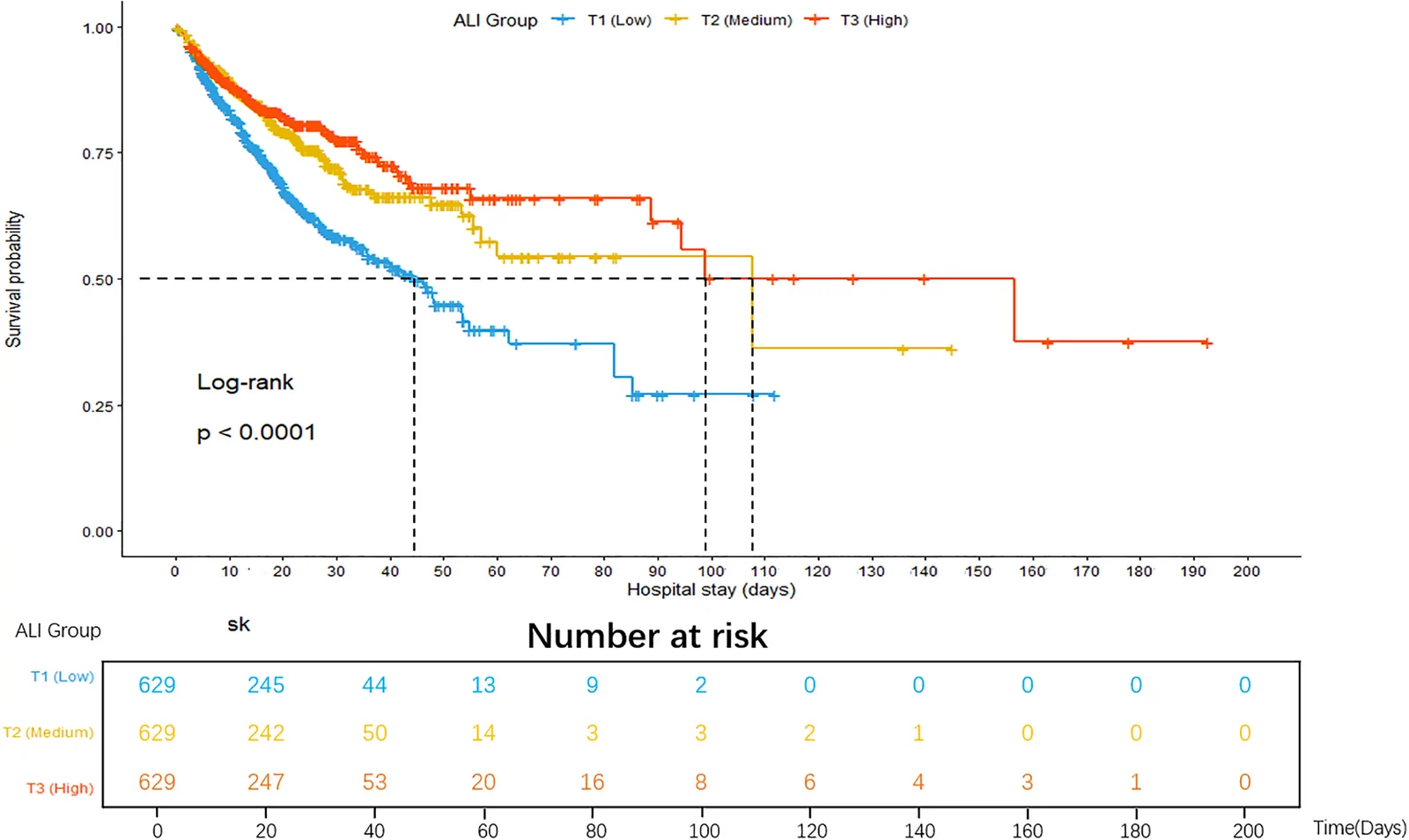
Kaplan-Meier survival curves for in-hospital mortality across the ALI tertiles. ALI, advanced lung cancer inflammation index.

To further evaluate the independent prognostic value of ALI, four multivariate Cox proportional hazards models were constructed with progressive adjustment for confounders (Table 2). In the unadjusted model, ln(ALI) as a continuous variable was inversely associated with in-hospital mortality (HR = 0.79, 95% CI 0.73–0.86; p < 0.001). After full adjustment for age, sex, SOFA score, and CCI (Model 4), ln(ALI) remained significantly associated with in-hospital mortality (HR = 0.86, 95% CI: 0.79–0.93; p < 0.001). When ALI was analyzed as tertiles, patients in the highest Tertile (T3) had a lower risk of in-hospital mortality than those in the lowest Tertile (T1) after full adjustment (HR = 0.63, 95% CI 0.52–0.77; p < 0.001; P for trend = 0.022). However, in the fully adjusted model that additionally incorporated KDIGO stage, urine output/oliguria, RRT, mechanical ventilation, vasopressor use, lactate, fluid balance, and nutritional support (n = 1218), the association between ln(ALI) and in-hospital mortality was attenuated and no longer statistically significant (HR = 0.94, 95% CI 0.86–1.04; p = 0.24), whereas lactate (HR = 1.11, 95% CI 1.09–1.14; p < 0.001), KDIGO stage (HR = 1.23, 95% CI 1.00–1.51; p = 0.046), and oliguria (HR = 1.31, 95% CI 1.01–1.70; p = 0.042) remained independent predictors.

### Dose-response relationship between ALI and in-hospital mortality

Restricted cubic spline analysis showed an approximately linear inverse association between ALI and in-hospital mortality after adjustment using Model 4. The p-value for nonlinearity was 0.438, indicating no significant departure from linearity (Fig. 2). The linearity of this association was consistent across prespecified subgroups, although the limited sample size in some strata may preclude detection of non-linear effects in specific populations.

**Fig. 2:**
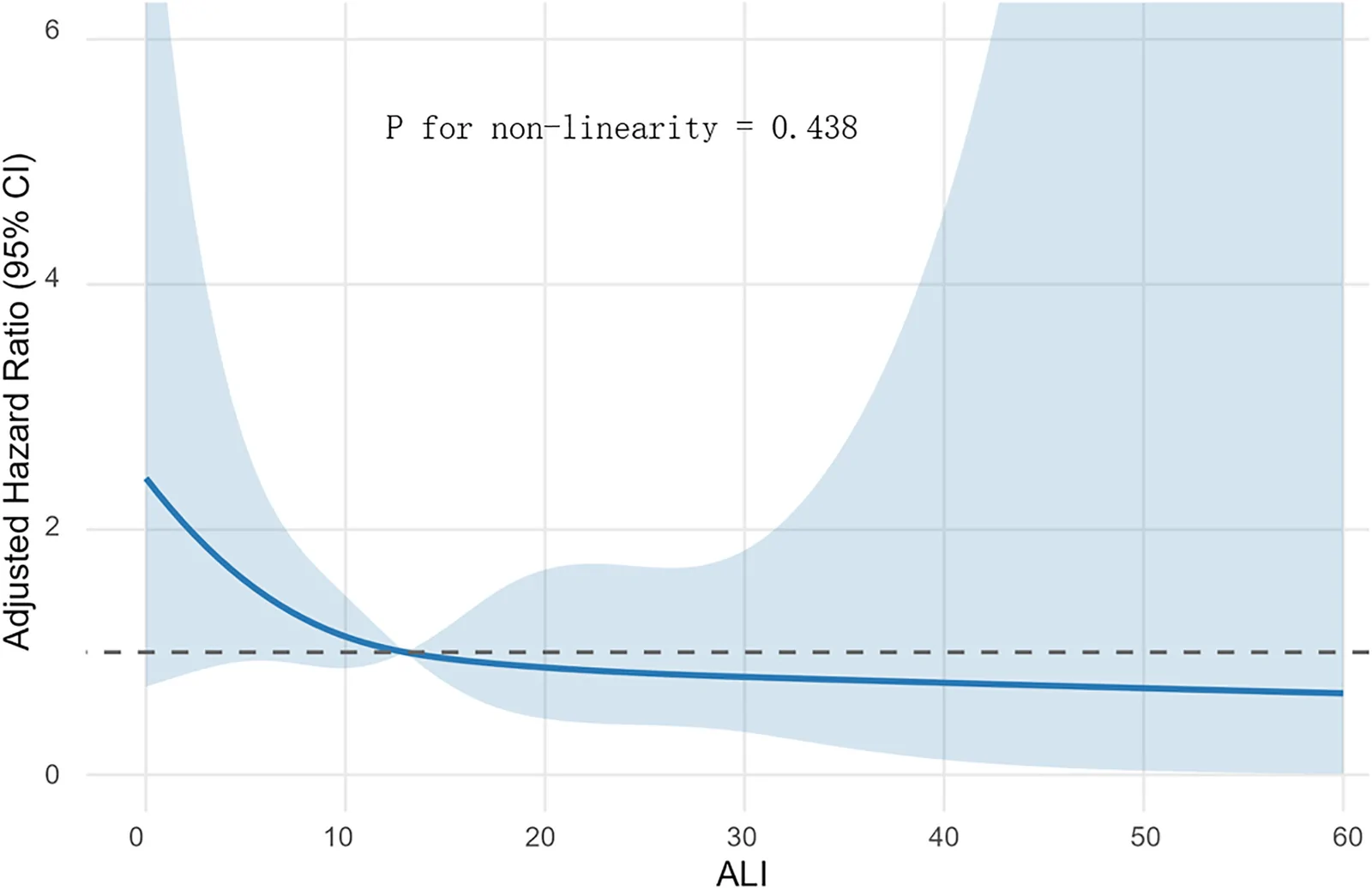
Restricted cubic spline analysis of the association between ALI and in-hospital mortality. ALI, advanced lung cancer inflammation index. The solid line and light blue area represent the estimated hazard ratio and corresponding 95% Confidence Interval. CI, Confidence Interval.

### Subgroup analysis for in-hospital mortality

Subgroup analyses showed no significant interaction by age, sex, BMI, hypertension, diabetes, or hyperlipidemia (all P for interaction > 0.05), suggesting that the association between ALI and in-hospital mortality did not materially differ across these prespecified subgroups (Fig. 3).

**Fig. 3:**
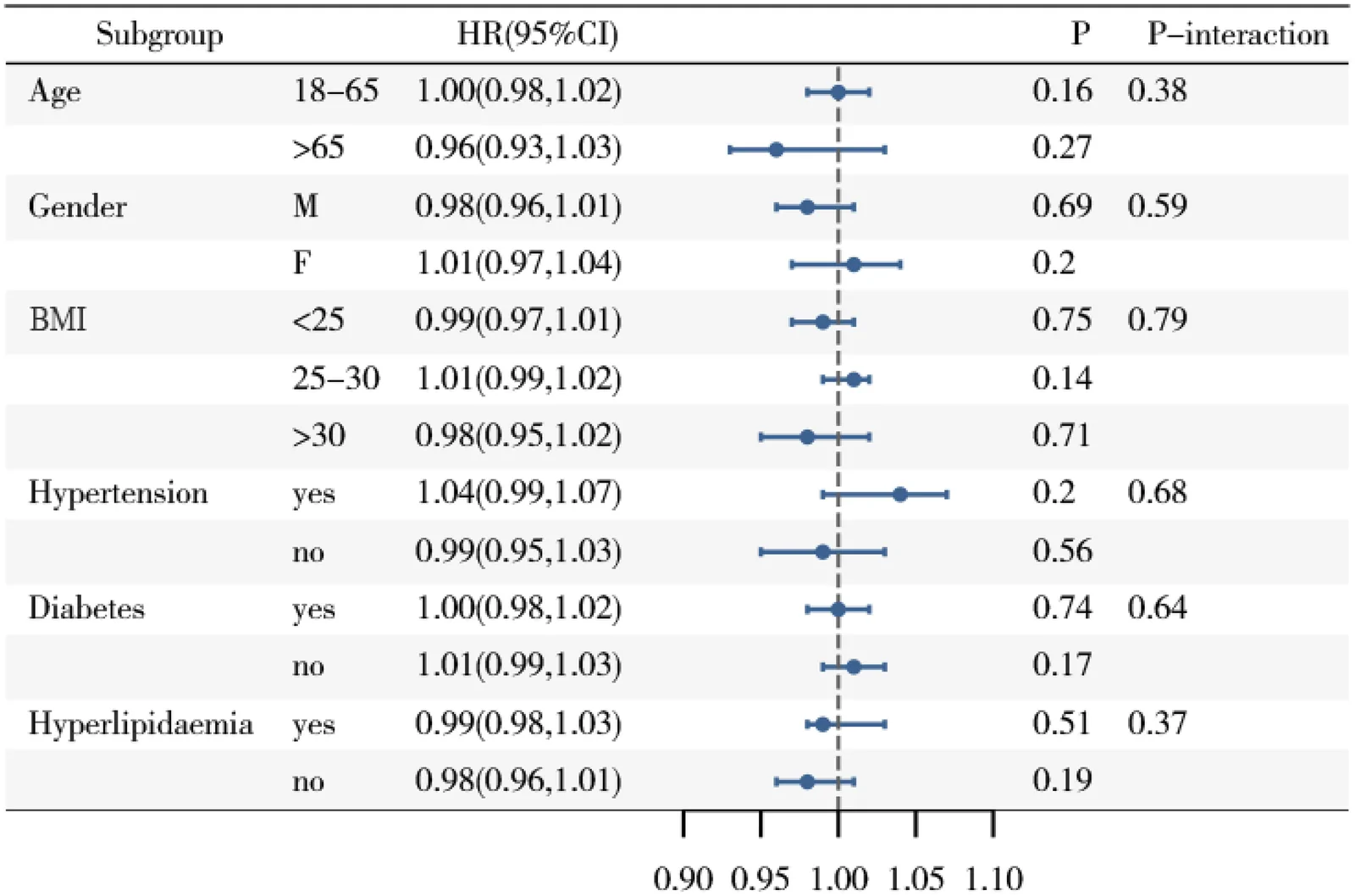
Association between ALI and in-hospital mortality according to baseline characteristics. CI, Confidence Interval; HR, Hazard Ratio; BMI, Body Mass Index; F, Female; M, Male.

### Association between ALI and 90-day mortality

We further evaluated the association between ALI and 90-day mortality in critically ill patients with AKI. Kaplan-Meier analysis showed that patients in the lowest ALI tertile had the highest cumulative 90-day mortality (log-rank p < 0.0001; Fig. 4). Univariate Cox regression analysis for 90-day mortality was performed (Supplementary Table 3). Higher SOFA score (HR = 1.10, 95% CI 1.08–1.13; p < 0.001) and lower serum albumin (HR = 0.85, 95% CI 0.75–0.96; p = 0.010) were associated with higher mortality risk. Higher CCI was associated with higher 90-day mortality in the univariate analysis (HR = 1.12, 95% CI 1.09–1.15; p < 0.001), consistent with the expected direction of effect. Multivariable Cox models were constructed to evaluate the independent association between ALI and 90-day mortality (Table 3). In the unadjusted model, ln(ALI) as a continuous variable was associated with lower 90-day mortality risk (HR = 0.74, 95% CI 0.68–0.79; p < 0.001). After full adjustment for age, sex, SOFA score, and CCI (Model 4), ln(ALI) remained significantly associated with 90-day mortality (HR = 0.81, 95% CI 0.75–0.87; p < 0.001). When analyzed as tertiles, patients in the highest ALI tertile (T3) had a lower risk of 90-day mortality than those in the lowest tertile (T1) after full adjustment (HR = 0.61, 95% CI 0.51–0.73; p = 0.026; P for trend = 0.014). In the fully adjusted model incorporating the newly extracted clinical variables (n = 1218), the association between ln(ALI) and 90-day mortality was attenuated but remained statistically significant (HR = 0.91, 95% CI 0.83–0.99; p = 0.023). Lactate (HR=1.10, 95% CI 1.08–1.13; p < 0.001), KDIGO stage (HR = 1.31, 95% CI 1.09–1.56; p = 0.003), and oliguria (HR = 1.36, 95% CI 1.08–1.70; p = 0.008) were independent predictors of 90-day mortality.

**Fig. 4:**
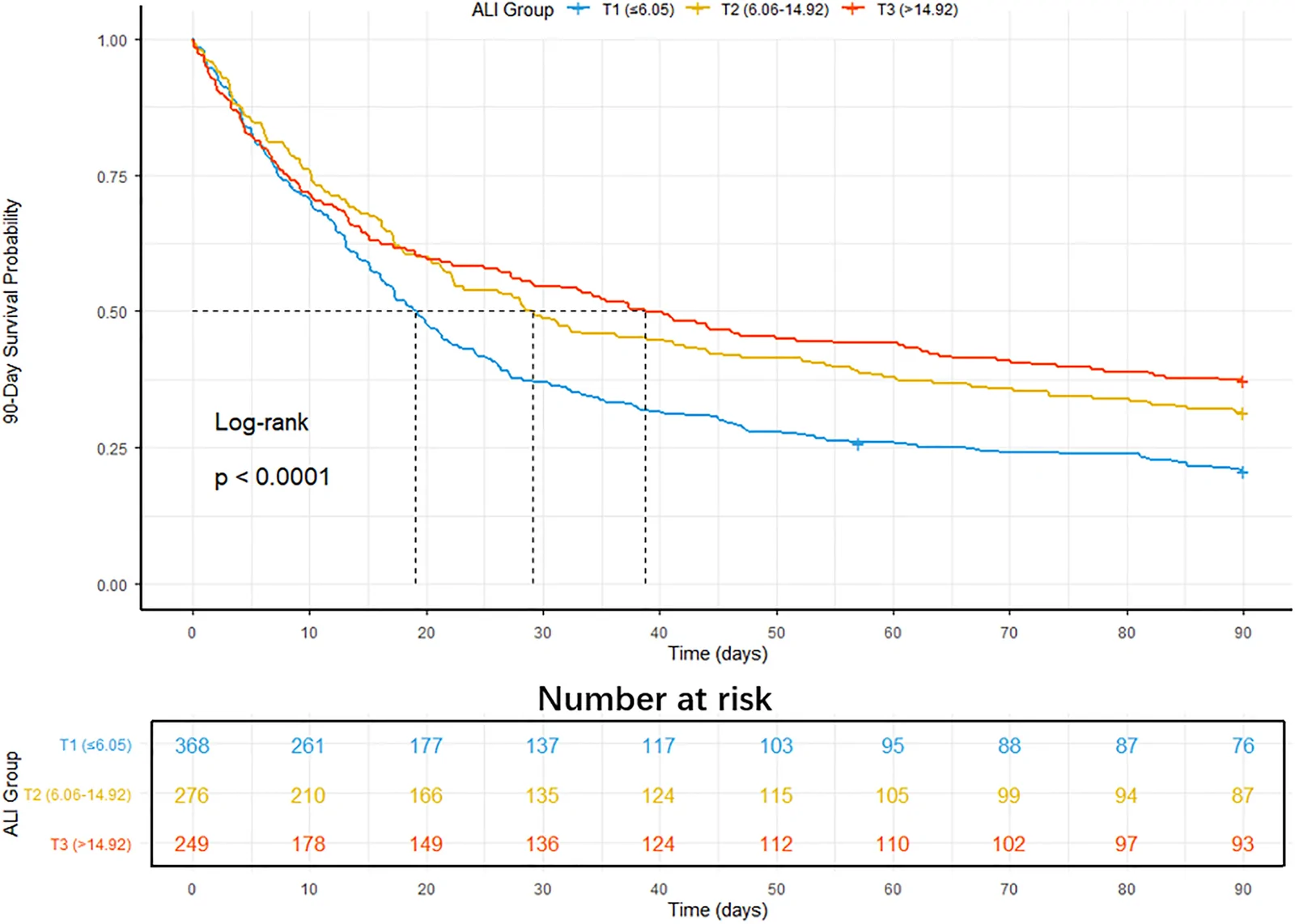
Kaplan-Meier survival curves for 90-day mortality across the ALI tertiles. ALI, Advanced Lung cancer Inflammation index.

**Table 3:** Association between ALI and 90-day mortality.

Model		ln(ALI)	ALI tertiles			P for trend
			T1	T2	T3	
Model 1	HR (95% CI)	0.74 (0.68‒0.79)	Reference	0.57 (0.48‒0.69)	0.46 (0.38‒0.56)	<0.001
	p	<0.001		<0.001	<0.001	
Model 2	HR (95% CI)	0.75 (0.69‒0.80)	Reference	0.58 (0.48‒0.69)	0.48 (0.39‒0.58)	<0.001
	p	<0.001		<0.001	<0.001	
Model 3	HR (95% CI)	0.95 (0.87‒1.03)	Reference	0.84 (0.67‒1.04)	0.83 (0.65‒1.05)	0.085
	p	0.203		0.105	0.114	
Model 4	HR (95% CI)	0.94 (0.86‒1.03)	Reference	0.84 (0.67‒1.04)	0.84 (0.66‒1.06)	0.102
	p	0.173		0.109	0.136	

ALI, Advanced Lung Cancer Inflammation Index; CI, Confidence Interval; HR, Hazard Ratio; ln, Natural logarithm.

Model 1: Unadjusted.

Model 2: Adjusted for age and sex.

Model 3: Model 2 + SOFA score, Charlson Comorbidity Index, lactate, KDIGO stage, oliguria, heart rate, glucose, blood urea nitrogen, and white blood cell count.

Model 4: Model 3 + RRT, mechanical ventilation, vasopressor use, fluid balance, and nutritional support.

### Incremental prognostic value of ALI

To evaluate whether ALI adds incremental prognostic value beyond established predictors, we compared model discrimination and reclassification (Supplementary Table 4). For 90-day mortality, adding ln(ALI) to the base model (age, sex, SOFA, CCI) increased the C-statistic from 0.669 to 0.683 (ΔC = 0.014) with an NRI of 0.054 and an IDI of 0.088; for in-hospital mortality, the C-statistic increased from 0.684 to 0.691 (ΔC = 0.006) with an NRI of 0.022 and an IDI of 0.037. When each individual marker was added to the base model, ln(ALI) (C-index 0.683) performed similarly to NLR (C-index 0.680) and only marginally better than albumin (C-index 0.676) or BMI (C-index 0.672) for 90-day mortality. These results indicate that ALI provides only modest incremental discrimination beyond established predictors and does not clearly outperform the simpler NLR alone.

## Discussion

In this retrospective cohort study of 1887 critically ill patients with AKI from the MIMIC-IV database, lower ALI at ICU admission was associated with higher in-hospital and 90-day mortality in unadjusted and partially adjusted models. The inverse association between ALI and in-hospital mortality was approximately linear; however, this association was substantially attenuated and no longer statistically significant after full adjustment for disease-severity and organ-support variables, indicating that much of the unadjusted association is explained by illness severity rather than by ALI per se. Subgroup analyses did not detect significant effect modification by age, sex, BMI, hypertension, diabetes, or hyperlipidemia, suggesting that the association was broadly similar across these prespecified clinical strata.

AKI in critically ill patients is closely linked to systemic inflammation, microcirculatory dysfunction, immune dysregulation, catabolism, and nutritional depletion. Recent studies have suggested that systemic inflammatory indices may help identify patients with sepsis-associated AKI who are at high risk of death.^19^^,^^20^ Maeda et al. demonstrated that lower ALI predicted worse long-term outcomes in patients with acute decompensated heart failure,^21^ and ALI has also been studied in sepsis-associated AKI.^16^ Our findings are biologically plausible because ALI integrates three routinely available markers: BMI, reflecting body mass and energy reserves; serum albumin, reflecting both nutritional status and inflammatory burden; and NLR, reflecting systemic inflammation and immune imbalance. These components may capture complementary dimensions of risk in critically ill patients with AKI.

Malnutrition and inflammation are common in patients with AKI, and this malnutrition–inflammation complex is associated with adverse outcomes.^22^, ^23^ In critical illness, intestinal edema, hepatic dysfunction, cytokine-driven hypercatabolism, insulin resistance, fluid accumulation, and reduced nutritional intake may worsen nutritional status and amplify inflammatory responses. Serum albumin is frequently used as a marker of nutritional and inflammatory status and has been associated with mortality in AKI and other critical illnesses.^24^^,^^25^ BMI has a complex association with outcomes in AKI, with prior studies reporting J-shaped, U-shaped, linear, or obesity-paradox patterns.^26^^,^^27^ NLR has also been reported as a potential biomarker for AKI prediction and risk stratification.^28^ By combining BMI, albumin, and NLR, ALI may better reflect the coexistence of malnutrition and inflammation than any single component alone.

Li et al. evaluated ALI in patients with sepsis-associated AKI from the MIMIC-IV database and reported that lower ALI was associated with higher 28-day and 90-day mortality.^16^ Importantly, there is no cohort or temporal overlap between our study and that of Li et al.: our cohort included all critically ill AKI patients rather than the sepsis-AKI subgroup, used different inclusion/exclusion criteria, and analyzed different primary outcomes (in-hospital mortality as the primary endpoint). The approximately linear association observed in our in-hospital mortality analysis may differ from findings in sepsis-associated AKI because septic AKI is characterized by more pronounced systemic inflammation and may have different threshold effects. Nevertheless, because AKI etiology, AKI stage, urine output, and renal replacement therapy were not fully captured in the current analysis, the clinical generalizability of ALI across AKI subtypes should be interpreted cautiously and validated in future studies. Future studies should quantify the proportion of septic versus non-septic AKI and examine the ALI-mortality association within these etiological subgroups, as the inflammatory milieu and nutritional trajectory may differ substantially between septic and non-septic AKI.

ALI was originally developed for non-small cell lung cancer patients, and its application to AKI and other non-cancer critical illnesses is relatively recent. While ALI has been studied in heart failure,^14^ type 2 diabetes,^15^ and sepsis-associated AKI,^16^ it has not been validated in broader AKI populations beyond the MIMIC-IV database. The optimal cutoff values for ALI may differ across clinical contexts, and the tertile-based cutoffs identified in this cohort (T1 < 6.05, T2: 6.05–14.92, T3 ≥ 14.92) may not be directly applicable to other populations. External validation in geographically and demographically diverse cohorts is essential before widespread clinical adoption. A recent study by Sahan et al.^29^ highlighted the importance of critical appraisal when applying composite inflammatory indices in acute care settings. Consistent with recent methodological studies in MIMIC-IV cohorts emphasizing rigorous evaluation of composite biomarkers,^30^, ^31^, ^32^ our restricted cubic spline analysis confirmed an approximately linear association (P for nonlinearity = 0.438) with no significant subgroup heterogeneity, further supporting a tempered interpretation of ALI's prognostic role.

Several limitations should be acknowledged. First, as a retrospective observational study, causal inference cannot be established and residual confounding may remain despite multivariable adjustment; factors such as frailty, nutritional intake, and the timing and cause of AKI were not fully captured. Second, AKI was identified using ICD codes rather than formal KDIGO criteria, which may introduce misclassification; we approximated and adjusted for KDIGO stage, but this approximation is imperfect. Third, excluding patients with short ICU stays or missing data may have introduced selection bias; median imputation restored the full sample and the ALI-mortality association re-emerged, suggesting our complete-case findings may be limited by sample size, and formal multiple imputation is warranted. Fourth, as a single-center analysis, generalizability is limited, and the study supports an association but does not establish clinical utility; prospective multicenter external validation is needed before ALI can be recommended for risk stratification.

## Conclusions

Lower ALI at ICU admission was associated with higher short-term mortality in critically ill patients with AKI, but this association was attenuated and no longer statistically significant after full adjustment for disease-severity markers and organ-support interventions, and was largely confined to non-septic AKI. This single-center retrospective study therefore supports an association but does not establish clinical utility; prospective multicenter external validation and formal assessment of incremental prognostic value are needed before ALI can be recommended for risk stratification.

## Supplementary material

Supplementary Material is available on the publisher's website along with the published article.

## Data availability

The data used in this study are available from the MIMIC-IV database (https://physionet.org/content/mimiciv/). Access requires completion of the required training course and a data use agreement. The SQL code used for data extraction is available from the corresponding authors upon reasonable request.

## Ethics approval and consent to participate

The use of the MIMIC-IV database was approved by the Institutional Review Board of the Massachusetts Institute of Technology (approval date: 14-Sep-2022; approval number: 51,474,658). The database contains de-identified public data, and all users are required to complete data-use training before access. Therefore, additional informed consent was waived for this study.

## Human and animal rights

The study was performed according to the guidelines of the Helsinki Declaration.

## Authors’ contributions

The authors confirm their contribution to the paper as follows: B.F.L. and Y.X. designed the study. Y.C.Z. collected, analyzed, and interpreted the data and prepared the figures and tables. Q.Q.L. and Y.X. drafted the manuscript. Y.X. and B.F.L. revised the manuscript. All authors read and approved the final manuscript.

## Funding

None.

## Declaration of competing interest

The authors declare no conflicts of interest.
