# Supplementary material for: Advanced lung cancer inflammation index is associated with mortality in critically ill patients with acute kidney injury
Source: Clinics (Sao Paulo). 2026 Sep 19;81:101156. doi: 10.1016/j.clinsp.2026.101156 (PMC13628232; doi:10.1016/j.clinsp.2026.101156)
Supplement: Supplementary file 1 [file mmc1.docx]

**CLINICS-D-26-01375_Supplementary Material**

**Supplementary Table 1** Risk of bias assessment using the ROBINS-I tool.

| **ROBINS-I domain** | **Risk of bias** |
| --- | --- |
| Bias due to confounding | Serious |
| Bias in selection of participants | Moderate |
| Bias in classification of interventions | Low |
| Bias due to deviations from intended interventions | Low |
| Bias due to missing data | Moderate |
| Bias in measurement of outcomes | Low |
| Bias in selection of the reported result | Low |
| Overall | Moderate |

ROBINS-I, Risk Of Bias In Non-randomized Studies of Interventions.

**Supplementary Table 2** Univariate Cox regression analysis for in-hospital mortality.

| **Variable** | **HR (95% CI)** | **p** |
| --- | --- | --- |
| Age | 1.01 (1.01‒1.02) | <0.001 |
| Gender (male) | 0.86 (0.71‒1.03) | 0.096 |
| Heart rate | 1.01 (1.00‒1.01) | 0.001 |
| SpO2 | 0.89 (0.87‒0.91) | <0.001 |
| SBP | 0.98 (0.98‒0.99) | <0.001 |
| DBP | 0.98 (0.96‒0.99) | 0.007 |
| Hematocrit | 1.00 (0.99‒1.01) | 0.731 |
| Creatinine | 1.03 (0.98‒1.08) | 0.193 |
| Platelets | 1.00 (1.00‒1.00) | 0.059 |
| Hemoglobin | 0.98 (0.95‒1.02) | 0.280 |
| Erythrocyte | 0.91 (0.83‒1.01) | 0.071 |
| MCV | 1.03 (1.02‒1.04) | <0.001 |
| MCH | 1.03 (1.00‒1.07) | 0.023 |
| MCHC | 0.91 (0.86‒0.96) | 0.001 |
| RDW | 1.10 (1.07‒1.13) | <0.001 |
| Leucocyte | 1.01 (1.00‒1.01) | 0.005 |
| Lymphocytes | 0.98 (0.97‒0.99) | 0.001 |
| Neutrophils | 1.00 (0.99‒1.01) | 0.632 |
| Monocytes | 0.98 (0.96‒1.00) | 0.070 |
| PT | 1.01 (1.01‒1.02) | <0.001 |
| INR | 1.18 (1.12‒1.25) | <0.001 |
| Glucose | 1.00 (1.00‒1.00) | 0.009 |
| BUN | 1.01 (1.00‒1.01) | <0.001 |
| Albumin | 0.79 (0.68‒0.91) | 0.001 |
| Sodium | 1.01 (0.99‒1.02) | 0.369 |
| Potassium | 1.03 (0.93‒1.15) | 0.532 |
| Calcium | 1.03 (0.93‒1.13) | 0.632 |
| ALT | 1.00 (1.00‒1.00) | <0.001 |
| AST | 1.00 (1.00‒1.00) | <0.001 |
| ALP | 1.00 (1.00‒1.00) | <0.001 |
| Total bilirubin | 1.03 (1.01‒1.04) | <0.001 |
| Hypertension | 0.92 (0.76‒1.11) | 0.388 |
| Diabetes mellitus type 2 | 0.94 (0.78‒1.13) | 0.502 |
| Diabetes mellitus type 1 | 0.27 (0.07‒1.06) | 0.061 |
| Myocardial infarct | 1.37 (1.10‒1.69) | 0.004 |
| Cancer | 1.53 (1.20‒1.95) | <0.001 |
| Hyperlipidaemia | 0.80 (0.66‒0.97) | 0.021 |
| Depression | 0.72 (0.53‒0.97) | 0.032 |
| Liver disease | 0.99 (0.53‒1.86) | 0.980 |
| Chronic pulmonary disease | 0.81 (0.52‒1.26) | 0.351 |
| Rheumatic disease | 0.79 (0.56‒1.11) | 0.176 |
| Peripheral vascular disease | 0.92 (0.56‒1.49) | 0.720 |
| Iron deficiency anemia | 1.11 (0.74‒1.68) | 0.616 |
| SOFA score | 1.14 (1.11‒1.16) | <0.001 |
| Charlson comorbidity index | 1.07 (1.04‒1.10) | <0.001 |
| BMI | 0.99 (0.98‒1.00) | 0.127 |
| NLR | 1.01 (1.01‒1.02) | <0.001 |
| Lactate | 1.13 (1.11‒1.15) | <0.001 |
| Oliguria | 2.13 (1.78‒2.55) | <0.001 |
| Renal replacement therapy | 2.19 (1.73‒2.79) | <0.001 |
| Mechanical ventilation | 1.17 (0.97‒1.41) | 0.097 |
| Vasopressor use | 1.55 (1.28‒1.87) | <0.001 |
| Fluid balance (24 h) | 1.00 (1.00‒1.00) | 0.007 |
| Nutritional support | 1.00 (0.64‒1.55) | 0.994 |
| KDIGO stage | 1.71 (1.49‒1.97) | <0.001 |

HR, Hazard Ratio; CI, Confidence Interval; RRT, Renal Replacement Therapy; KDIGO, Kidney Disease: Improving Global Outcomes.

**Supplementary Table 3** Univariate Cox regression analysis for 90-day mortality.

| **Variable** | **HR (95% CI)** | **p** |
| --- | --- | --- |
| Age | 1.02 (1.01‒1.03) | <0.001 |
| Gender (male) | 0.85 (0.72‒0.99) | 0.037 |
| Heart rate | 1.01 (1.00‒1.01) | 0.002 |
| SpO_2_ | 0.90 (0.88‒0.93) | <0.001 |
| SBP | 0.99 (0.98‒1.00) | 0.041 |
| DBP | 0.98 (0.97‒0.99) | 0.006 |
| Hematocrit | 0.99 (0.98‒1.00) | 0.011 |
| Creatinine | 1.04 (1.01‒1.08) | 0.017 |
| Platelets | 1.00 (1.00‒1.00) | 0.289 |
| Hemoglobin | 0.94 (0.92‒0.97) | <0.001 |
| Erythrocyte | 0.83 (0.76‒0.91) | <0.001 |
| MCV | 1.03 (1.02‒1.04) | <0.001 |
| MCH | 1.03 (1.00‒1.06) | 0.028 |
| MCHC | 0.89 (0.85‒0.93) | <0.001 |
| RDW | 1.12 (1.10‒1.15) | <0.001 |
| Leucocyte | 1.01 (1.00‒1.01) | 0.002 |
| Lymphocytes | 0.97 (0.96‒0.98) | <0.001 |
| Neutrophils | 1.00 (1.00‒1.01) | 0.262 |
| Monocytes | 0.99 (0.97‒1.01) | 0.243 |
| PT | 1.01 (1.01‒1.02) | <0.001 |
| INR | 1.19 (1.13‒1.25) | <0.001 |
| Glucose | 1.00 (1.00‒1.00) | <0.001 |
| BUN | 1.01 (1.01‒1.01) | <0.001 |
| Albumin | 0.63 (0.56‒0.72) | <0.001 |
| Sodium | 1.00 (0.99‒1.01) | 0.830 |
| Potassium | 1.11 (1.01‒1.21) | 0.026 |
| Calcium | 1.01 (0.92‒1.11) | 0.825 |
| ALT | 1.00 (1.00‒1.00) | 0.017 |
| AST | 1.00 (1.00‒1.00) | <0.001 |
| ALP | 1.00 (1.00‒1.00) | <0.001 |
| Total bilirubin | 1.03 (1.02‒1.05) | <0.001 |
| Hypertension | 0.96 (0.81‒1.13) | 0.614 |
| Diabetes mellitus type 2 | 0.96 (0.82‒1.13) | 0.631 |
| Diabetes mellitus type 1 | 0.39 (0.16‒0.95) | 0.038 |
| Myocardial infarct | 1.22 (1.02‒1.47) | 0.033 |
| Cancer | 1.77 (1.46‒2.14) | <0.001 |
| Hyperlipidaemia | 0.77 (0.66‒0.90) | 0.001 |
| Depression | 0.66 (0.51‒0.86) | 0.002 |
| Liver disease | 0.86 (0.46‒1.61) | 0.648 |
| Chronic pulmonary disease | 0.88 (0.60‒1.27) | 0.490 |
| Rheumatic disease | 0.94 (0.71‒1.23) | 0.643 |
| Peripheral vascular disease | 0.92 (0.60‒1.40) | 0.688 |
| Iron deficiency anemia | 1.11 (0.79‒1.56) | 0.558 |
| SOFA score | 1.13 (1.11‒1.16) | <0.001 |
| Charlson comorbidity index | 1.12 (1.09‒1.15) | <0.001 |
| BMI | 0.98 (0.97‒0.99) | <0.001 |
| NLR | 1.01 (1.01‒1.02) | <0.001 |
| Lactate | 1.13 (1.11‒1.15) | <0.001 |
| Oliguria | 2.18 (1.87‒2.55) | <0.001 |
| Renal replacement therapy | 2.44 (1.95‒3.05) | <0.001 |
| Mechanical ventilation | 1.14 (0.97‒1.33) | 0.118 |
| Vasopressor use | 1.40 (1.19‒1.64) | <0.001 |
| Fluid balance (24 h) | 1.00 (1.00‒1.00) | 0.011 |
| Nutritional support | 1.22 (0.81‒1.84) | 0.336 |
| KDIGO stage | 1.79 (1.58‒2.02) | <0.001 |

HR, Hazard Ratio; CI, Confidence Interval; RRT, Renal Replacement Therapy; KDIGO, Kidney Disease: Improving Global Outcomes.

**Supplementary Table 4** Incremental prognostic value of ALI beyond established predictors.

| **Model** | **C-statistic** | **ΔC** | **NRI** | **IDI** |
| --- | --- | --- | --- | --- |
| Panel A. Incremental value of adding ln(ALI) to the base model (age, sex, SOFA, CCI) |  |  |  |  |
| 90-day mortality, base model | 0.669 | Reference |  |  |
| 90-day mortality, base + ln(ALI) | 0.683 | 0.014 | 0.054 | 0.088 |
| In-hospital mortality, base model | 0.684 | Reference |  |  |
| In-hospital mortality, base + ln(ALI) | 0.691 | 0.006 | 0.022 | 0.037 |
| Panel B. C-statistic of base model with each marker added (90-day mortality) |  |  |  |  |
| Base + ln(ALI) | 0.683 |  |  |  |
| Base + NLR | 0.680 |  |  |  |
| Base + albumin | 0.676 |  |  |  |
| Base + BMI | 0.672 |  |  |  |

ALI, Advanced Lung Cancer Inflammation Index; BMI, Body Mass Index; CCI, Charlson Comorbidity Index; IDI, Integrated Discrimination Improvement; ln, natural logarithm; NLR, Neutrophil-to-Lymphocyte Ratio; NRI, Net Reclassification Improvement; SOFA, Sequential Organ Failure Assessment.

**Figure S1** Flow chart of the screening and enrolment of study participants.

**Figure S2** Comparison of ALI between patients with and without short-term mortality. (A) In-hospital mortality; (B) 90-day mortality. ALI, Advanced Lung cancer Inflammation index.
